# Characteristics and Risk Factors of Intraoperative Hypothermia in Adults: A Multicenter Prospective Observational Clinical Study

**DOI:** 10.3390/jcm15010031

**Published:** 2025-12-20

**Authors:** Hanqing Zhang, Xinglian Gao, Wen Ke, Zengyan Wang, Qiong Ma, Wenjing Yu, Juanjuan Hu, on behalf of the Intraoperative Hypothermia Investigators (12-Center Consortium)

**Affiliations:** 1 Union Hospital, Tongji Medical College, Huazhong University of Science and Technology, Wuhan 430000, China; 2 School of Nursing, Tongji Medical College, Huazhong University of Science and Technology, Wuhan 430030, China

**Keywords:** adults, intraoperative hypothermia, occurrence characteristics, risk factors, perioperative temperature management

## Abstract

**Objective:** Intraoperative hypothermia is a common perioperative complication. This large-scale, multicenter, prospective clinical study aimed to delineate the occurrence patterns of intraoperative hypothermia in adults and to identify its major independent risk factors, thereby providing evidence-based support for early clinical risk assessment and intervention. **Methods:** This study adopted a multicenter, prospective, observational design. Eligible participants were screened based on predefined inclusion and exclusion criteria, and a total of 4516 surgical patients (≥18 years) from 12 tertiary general hospitals across China were ultimately enrolled. Core body temperature was continuously monitored intraoperatively using standardized methods. Data on demographic characteristics, surgical and anesthesia-related parameters, and perioperative temperature management interventions were collected. Patients were stratified into groups according to the presence or absence of hypothermia (core temperature <36.0 °C). Univariate analyses were first conducted to identify associated factors, followed by multivariable logistic regression to determine factors independently associated with intraoperative hypothermia. **Results:** The overall incidence of intraoperative hypothermia among surgical patients was 23.82%. Hypothermia occurred most frequently in patients with a preoperative baseline core temperature ≤ 35.9 °C (85.93%). Among surgical specialties, hand surgery had the highest incidence of hypothermia (51.35%), and among surgical sites, procedures involving the upper extremities showed the highest rate (35.00%). Multivariable logistic regression analysis identified the following as independent risk factors for intraoperative hypothermia: Type of anesthesia (OR = 1.743, 95% CI: 0.834–3.644), ASA classification (OR = 1.408, 95% CI: 1.197–1.657), Surgical approach (OR = 0.735, 95% CI: 0.577–0.936), Skin disinfection site (OR = 2.024, 95% CI: 1.534–2.670), Volume of cold intravenous fluids infused (mL) (OR = 1.365, 95% CI: 1.140–1.633), Volume of transfused blood (U) (OR = 1.116, 95% CI: 0.807–1.542), Intraoperative blood loss (mL) (OR = 1.252, 95% CI: 0.892–1.756), and Duration of surgery (hours) (OR = 2.014, 95% CI: 1.683–2.411). **Conclusions:** The incidence of intraoperative hypothermia in adults was relatively high at 23.82% and was observed to be associated with multiple modifiable perioperative factors. These findings support the need to strengthen risk assessment and implement individualized temperature management strategies in clinical practice, with the goal of reducing the risk of intraoperative hypothermia and improving perioperative safety and outcomes.

## 1. Introduction

Intraoperative hypothermia is defined as a decrease in core body temperature below 36.0 °C during surgery and is among the most common perioperative complications. Reports indicate that the incidence of intraoperative hypothermia in adult patients ranges from 20% to 70%, and some studies suggest that, in the absence of effective preventive measures, it may exceed 80% [1]. Hypothermia is not only common but also clinically significant. A large body of evidence has demonstrated that intraoperative hypothermia impairs coagulation function, increases intraoperative blood loss, raises the risk of surgical site infection by two- to threefold, prolongs anesthetic drug metabolism, and elevates the incidence of cardiovascular complications [2,3,4]. In addition, intraoperative hypothermia is associated with prolonged stay in the recovery room, increased healthcare costs, and, in severe cases, may even pose a threat to patient survival [5]. Therefore, effective prevention and management of intraoperative hypothermia have become critical issues in perioperative care.

Although several studies have investigated risk factors associated with intraoperative hypothermia, most have been single-center, retrospective studies with limited sample sizes, which restrict the generalizability and representativeness of their findings. Furthermore, existing literature has reported inconsistent risk factors across different regions of the country, various surgical specialties, and diverse levels of perioperative management. To date, robust, systematic, and prospective clinical evidence remains lacking.

Therefore, building upon previous single-center studies, we conducted a multicenter clinical investigation across 12 tertiary general hospitals in China, employing a prospective observational study design. The aim was to determine the incidence and clinical characteristics of intraoperative hypothermia in adults and to identify its major independent risk factors, thereby providing evidence-based support for the development of risk assessment systems and temperature management strategies.

## 2. Materials and Methods

This study was conducted in accordance with the Declaration of Helsinki and was approved by the Ethics Committee of Union Hospital, Tongji Medical College, Huazhong University of Science and Technology (Approval No. [2024] Lunshen Zi (0802-01)). The study was also registered in the public clinical trial registry (https://www.clinicaltrials.gov/study/NCT06836245?cond=NCT06836245&rank=1, accessed on 14 February 2025). All participants voluntarily enrolled after providing informed consent, and the study strictly adhered to the principles of voluntariness and confidentiality. To ensure consistency across all 12 participating hospitals, a unified study protocol was developed and implemented, covering inclusion/exclusion criteria, temperature-monitoring methods, instrument calibration procedures, and data-entry requirements. All centers were required to strictly follow this standardized protocol throughout the study period.

### 2.1. Study Design Period and Regions

This multicenter, prospective, observational study was conducted from November 2024 to February 2025. A total of 12 representative tertiary general hospitals across China were recruited, with geographic distribution covering major regions nationwide, including Central China, East China, Southwest, Northwest, South China, North China, and Northeast China. Because perioperative practices may vary among institutions, the coordinating center established a Standardized Operating Procedure that was uniformly disseminated to all participating hospitals. This explicitly defined the operational procedures for patient screening, perioperative temperature monitoring, and data-quality management. All hospitals were instructed to adhere to this unified framework to minimize inter-center variability and enhance comparability of results.

### 2.2. Study Population

Multicenter nursing staff participating in the clinical study were uniformly trained by the lead center. All staff held at least a bachelor’s degree (preferably postgraduate), had prior experience in clinical research, and possessed competencies in data collection and statistical analysis. Adult surgical patients were consecutively enrolled according to standardized criteria until each participating center achieved the predetermined sample size.

Inclusion criteria: (1) Surgical patients aged ≥18 years; (2) Patients informed of and consenting to routine intraoperative core temperature monitoring.

Exclusion criteria: (1) Patients undergoing surgery under local infiltration anesthesia; (2) Patients requiring intraoperative hypothermia therapy or receiving special warming interventions; (3) Patients with abnormal thermoregulatory mechanisms due to specific diseases; (4) Patients with incomplete intraoperative temperature records or missing relevant surgical data.

To ensure uniform application of inclusion and exclusion criteria across all centers, all investigators were required to follow the same eligibility checklist and to document screening results using a standardized form. The coordinating center regularly reviewed enrollment logs from each site to verify adherence to the unified recruitment protocol.

### 2.3. Study Variables

Dependent variable: Intraoperative core temperature < 36 °C.

Independent variables: (1) Sociodemographic and clinical characteristics: Age (years; 18–27, 28–37, 38–47, 48–57, 58–67, 68–77, 78–87, ≥88), Sex (male; female), Surgical specialty (otorhinolaryngology; orthopedics; obstetrics/gynecology; hepatobiliary surgery; thyroid/breast surgery; urology; thoracic surgery; gastrointestinal surgery; hand surgery; stomatology; general surgery; neurosurgery; vascular surgery; emergency surgery; cardiovascular surgery), BMI (kg/m^2^; <18.5, 18.5–24.9, 25.0–29.9, ≥30.0), Surgical site (head and neck; thoracic; abdominal; back; upper extremity; lower extremity; pelvis and perineum), Baseline core temperature (°C; ≤35.9, ≥36.0). (2) Intraoperative factors: Preoperative bowel preparation (none; oral laxatives; cleansing enema), Anesthesia type (regional; general; combined), ASA classification (I–II, III, ≥IV), Surgical approach (superficial/soft tissue; minimally invasive; open surgery), Operating room cleanliness (ten-thousand class; thousand class; hundred class), Skin disinfection site (head/neck; extremities; thorax/abdomen/back), Volume of intravenous cold fluid (mL; <1000, 1000~2000, >2000), Volume of cold irrigation fluid (mL; <1000, 1000~2000, >2000), Intraoperative allogeneic blood transfusion (U; <2, 2~4, >4), Intraoperative blood loss (mL; <400, 400~800, >800), and Surgical duration (h; <2, 2~4, >4).

### 2.4. Definition of Intraoperative Hypothermia

In this study, the gold standard definition of intraoperative hypothermia was: core temperature measured every 30 min from the initiation of anesthesia until the patient left the operating room, with a core temperature < 36 °C recorded on two or more occasions. Core temperature was defined as the value obtained from validated core-monitoring sites (distal esophagus, tympanic membrane, or nasopharynx, as specified in Section 2.6.1) using continuous electronic thermometry. The same site-specific measurement rules were applied consistently across all centers (distal esophageal probes under general anesthesia; tympanic or nasopharyngeal probes under regional or combined anesthesia).

### 2.5. Sample Size Calculation and Sampling Method

Sample size: According to statistical requirements for variable analysis, the sample size should be at least 5–10 times the number of scale items. The “Data Collection Form for Factors Associated with Intraoperative Hypothermia in Adults”, constructed by the research team, contained 11 items. To further enhance reliability and account for a 10% dropout rate, each study center was required to recruit no fewer than 61–121 patients, with a total sample size across the 12 hospitals of no less than 732–1452 cases. During the data collection period, a total of 4516 valid cases were ultimately obtained.

Sampling method: Prospective data collection was performed using a convenience sampling approach.

### 2.6. Data Collection

#### 2.6.1. Survey Instruments

In this study, the “Data Collection Form for Factors Associated with Intraoperative Hypothermia in Adults” was developed through a comprehensive review of domestic and international literature, clinical guidelines, and expert consultation. (1) General patient information form: Records included age, medical record number, sex, surgical department, surgical name, surgical site, duration of surgery, and intraoperative nursing measures for hypothermia. (2) Intraoperative core temperature monitoring form: Designed to dynamically monitor intraoperative changes in patients’ core temperature, with recordings taken every 30 min from entry into the operating room until completion of surgery and departure. This form allowed documentation from six potential core temperature monitoring sites: distal esophagus, tympanic membrane, nasopharynx, bladder, rectum, and pulmonary artery. In this study, three sites were actually used for intraoperative measurement: distal esophagus, tympanic membrane, and nasopharynx. For patients under general anesthesia, core temperature was measured using a distal esophageal probe, while for those receiving regional or combined anesthesia, tympanic or nasopharyngeal measurements were selected based on clinical feasibility. Across all centers, the same site-selection (distal esophagus for general anesthesia; tympanic or nasopharyngeal sites for regional or combined anesthesia) was applied according to a unified protocol, so that the choice of monitoring site reflected anesthesia technique rather than all centers’ preference. All temperature values were obtained using continuous electronic thermometry with disposable probes integrated into the anesthesia monitoring system. All centers used standardized temperature-monitoring devices that were pre-calibrated and verified before each operation to ensure measurement accuracy within ±0.1 °C. A uniform standardized measurement protocol was implemented consistently across all participating hospitals to ensure inter-center comparability and data reliability. (3) Adult Intraoperative Hypothermia Risk Assessment Scale [6]: Developed by the research team, this scale demonstrated good reliability and validity, with a Cronbach’s α coefficient of 0.790 and an area under the ROC curve (AUC) of 0.800. The scale consisted of 11 risk factor items, including preoperative bowel preparation, anesthesia type, ASA classification, surgical approach, operating room cleanliness level, site of skin disinfection, volume of cold intravenous fluid infusion, intraoperative cold irrigation volume, intraoperative allogeneic blood transfusion, intraoperative blood loss, and surgical duration. Each item was scored on a scale of 1–3, with a total score ranging from 11 to 36. The evaluation covered the entire period from patient entry into the operating room until the end of surgery, and each item was quantitatively rated by the investigator.

To minimize institutional variation in temperature-monitoring techniques, all centers used standardized temperature-monitoring devices of the same model, and each device underwent calibration and verification before every operation according to a unified protocol. A detailed measurement guideline was provided to all centers. Furthermore, all centers were required to record temperature using an identical data-collection form to ensure inter-center comparability. Although the three core-monitoring sites used in this study (distal esophagus, tympanic membrane, and nasopharynx) are all widely accepted as reliable surrogates of core temperature, small systematic differences between sites may still exist and could have contributed to minor residual variability in absolute temperature values. However, the use of a standardized protocol and uniform monitoring equipment across all centers was intended to minimize such variability and support consistency of the findings.

#### 2.6.2. Survey Method

Prior to study initiation, a two-week standardized training program was provided for the principal investigators of the 12 participating hospitals. The training covered knowledge related to intraoperative hypothermia, risk factor assessment, standardized use of the Adult Intraoperative Hypothermia Risk Assessment Scale, and techniques for core temperature monitoring. Both online and in-person modalities were employed to ensure a comprehensive understanding and mastery of study instruments and operational procedures. Following training, participants underwent an assessment on risk factor evaluation and scale application to guarantee consistency in evaluation and data collection across all centers. To facilitate communication, the research team established a dedicated group for the “Intraoperative Hypothermia Assessment Study in Patients,” enabling timely interaction between centers and ensuring the quality and completeness of data collection. Core temperature measurement time points included: (1) baseline core temperature in the preoperative waiting area; (2) at anesthesia induction; (3) at surgical incision; (4) every 30 min intraoperatively; (5) at the end of surgery. Informed consent was obtained from all surgical patients before assessment at each center, ensuring compliance with ethical standards.

In addition, to maintain consistency in evaluation and data-collection procedures, all investigators were required to pass a competency assessment on the use of the Adult Intraoperative Hypothermia Risk Assessment Scale and the temperature-monitoring protocol. The coordinating center established a real-time communication platform to address operational issues promptly across all centers. Periodic audits of randomly selected cases were conducted to ensure adherence to the standardized protocol.

### 2.7. Data Quality Control

All raw data were archived in paper form. Research materials were verified by two independent reviewers, organized, and then uniformly entered into an electronic database by trained researchers. After data entry, procedures for data cleaning and logical verification were performed to prevent duplicate entry or omissions. Records with obvious logical errors, missing assessment items, or incomplete information were excluded to ensure the authenticity and validity of the study data. All electronic data were restricted to use within this study and strictly adhered to data confidentiality and management regulations. To further strengthen inter-center data consistency, all centers were required to submit temperature-monitoring records and data sheets for validation. Any discrepancies identified during quality audits were communicated back to the respective centers to ensure uniform data integrity across all sites.

### 2.8. Data Processing and Statistical Analysis

All variables were entered, coded, and error-checked using EpiData software (version 4.6). Data were organized, entered, and double-checked with Excel 2024, and then centrally stored. All statistical analyses were performed using SPSS software (version 31.0; IBM Corp., Armonk, NY, USA). Descriptive statistics were conducted, with continuous variables expressed as mean ± standard deviation (SD), and between-group comparisons assessed using the independent-samples *t*-test. Categorical variables were expressed as frequencies and percentages (%), and between-group comparisons were conducted using the χ^2^ test. Univariate analysis was used to preliminarily screen potential risk factors associated with intraoperative hypothermia, with *p* < 0.05 considered statistically significant. Variables with significance in the univariate analysis were entered into multivariable logistic regression, with intraoperative hypothermia (core temperature < 36.0 °C) as the dependent variable. Odds ratios (ORs) and 95% confidence intervals (95% CIs) were calculated to identify independent risk factors. All statistical tests were two-tailed, and *p* < 0.05 was considered statistically significant.

## 3. Results

### 3.1. Study Population Screening

**Figure 1:**
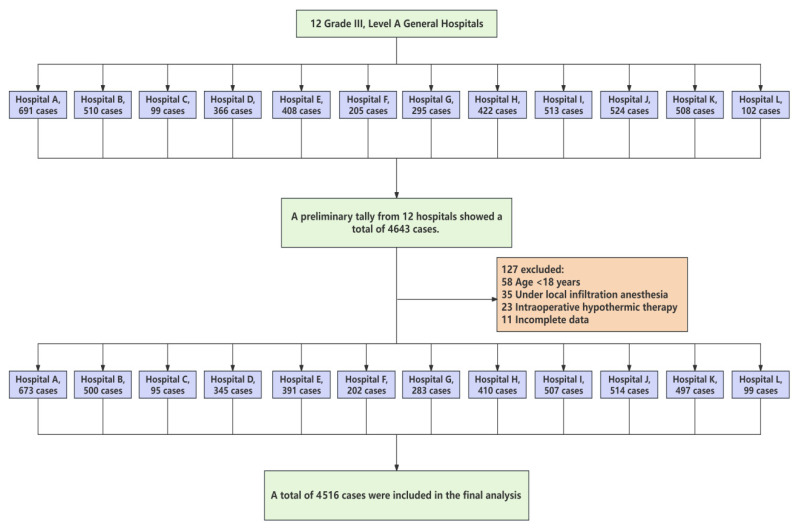
Flowchart of study population across 12 hospitals. Light green boxes indicate overall of case numbers at different stages of screening and inclusion. Light blue boxes represent each participating hospitals and corresponding number of cases. Light orange boxes denote excluded cases and reasons for exclusion.

A total of 4643 surgical patients were initially enrolled across 12 tertiary general hospitals in China. Based on the predefined inclusion and exclusion criteria, 127 patients were excluded: 58 patients were under 18 years of age, 35 underwent surgery under local infiltration anesthesia, 23 required intraoperative therapeutic hypothermia, and 11 had incomplete surgical data or temperature records. Ultimately, 4516 patients were included in the statistical analysis (Figure 1).

### 3.2. Characteristics of Intraoperative Hypothermia

**Figure 2:**
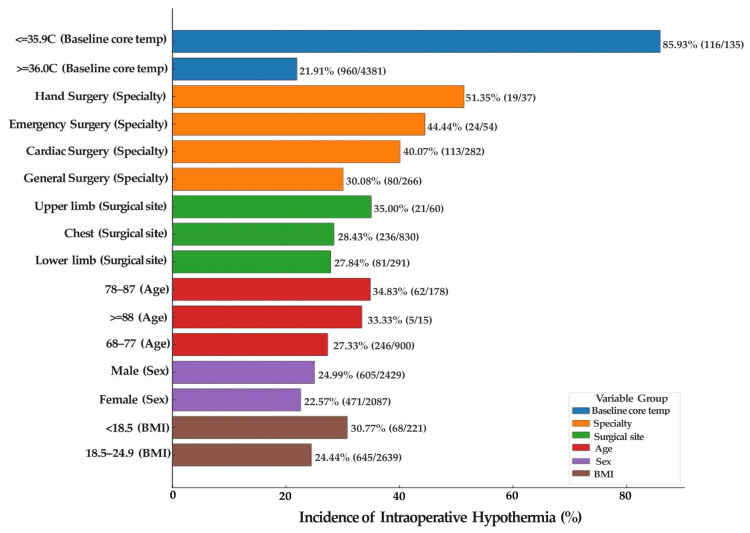
Incidence of intraoperative hypothermia across key stratified variables.

Among the 4516 surgical patients included in this study, 1076 developed intraoperative hypothermia, yielding an overall incidence of 23.82%. Patients with a baseline core temperature ≤ 35.9 °C had an incidence of 85.93%, which was significantly higher than that of patients with baseline core temperature ≥ 36.0 °C (21.91%). Marked variation in hypothermia incidence was observed across surgical specialties: hand surgery (51.35%), emergency surgery (44.44%), and cardiovascular surgery (40.07%) showed the highest rates, while otorhinolaryngology (13.92%) and obstetrics/gynecology (16.24%) had the lowest. By surgical site, upper extremity procedures (35.00%), thoracic surgery (28.43%), and lower extremity procedures (27.84%) demonstrated relatively higher incidence, whereas back surgery had the lowest (13.04%). An age-related trend was evident, with incidence increasing progressively from 16.74% in the 18–27-year group to 34.83% in the 78–87-year group. Males (24.99%) had a slightly higher incidence compared to females (22.57%). In BMI stratification, underweight patients (<18.5 kg/m^2^) had the highest incidence of hypothermia (30.77%), while obese patients (≥30.0 kg/m^2^) had the lowest (16.17%). The incidence of intraoperative hypothermia under different clinical characteristics is presented in Table 1, and key stratified variables are illustrated in Figure 2.

### 3.3. Intraoperative Core Temperature Changes Under Different Warming Interventions

**Figure 3:**
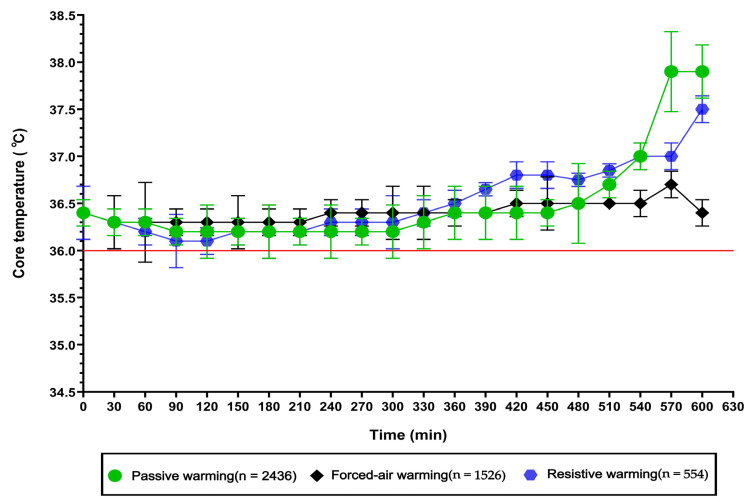
Changes in intraoperative core temperature.

Among the surgical patients included in this study, three main perioperative warming interventions were commonly applied: passive insulation (*n* = 2436), forced-air warming (*n* = 1526), and resistive heating (*n* = 554). At the beginning of surgery, the core temperature levels of the three groups were similar, generally maintained between 36.35–36.40 °C. During the early intraoperative period (0–210 min), core temperatures in all groups showed varying degrees of decline, with the most pronounced decrease observed in the passive insulation group, where some patients’ temperatures approached the hypothermia threshold (36.0 °C). As surgery extended beyond 240 min, patients in the forced-air warming and resistive heating groups exhibited gradual increases in core temperature, both maintaining higher levels than the passive insulation group. During the later stage of surgery (330–420 min), the resistive heating group demonstrated a faster rate of temperature recovery, gradually surpassing the forced-air warming group. In contrast, patients managed with passive insulation alone did not show a clear trend of intraoperative rewarming until after 450 min. The intraoperative core temperature change curves under the three warming interventions are shown in Figure 3. It should be noted that this study was observational in nature, and the choice of warming intervention was based on routine clinical practice. The selection of a specific warming method may have been influenced by surgical type, patient characteristics, or centers’ preferences. Therefore, the observed differences in intraoperative core temperature trajectories among the three groups should be reflected as clinical practice patterns. These findings indicate associations between warming strategies and intraoperative temperature changes, rather than definitive comparative effectiveness.

### 3.4. Univariate Analysis of Risk Factors for Intraoperative Hypothermia

To further investigate the risk factors associated with intraoperative hypothermia, univariate analyses were conducted for multiple perioperative variables. The results demonstrated significant associations between certain clinical and surgical factors and the occurrence of intraoperative hypothermia. Regarding patient baseline characteristics, ASA classification was significantly correlated with hypothermia incidence (χ^2^ = 55.850, *p* < 0.001). Higher ASA grades were associated with higher rates of hypothermia, indicating a statistical relationship. Among surgical factors, anesthesia type significantly affected hypothermia incidence (χ^2^ = 6.584, *p* = 0.037), with patients receiving general or combined anesthesia showing higher rates compared with those under regional anesthesia alone. Surgical approach was also a significant factor (χ^2^ = 11.143, *p* = 0.004), with open surgeries having a higher incidence than superficial or minimally invasive procedures. The site of skin disinfection showed significant differences (χ^2^ = 27.297, *p* < 0.001): surgeries involving the extremities, thorax, abdomen, and back had markedly higher hypothermia rates compared with head and neck surgeries. Surgical duration was strongly associated with hypothermia (χ^2^ = 177.803, *p* < 0.001), with longer procedures corresponding to a higher incidence. In fluid management, the volume of cold intravenous infusion was significantly associated with intraoperative hypothermia (χ^2^ = 77.862, *p* < 0.001). Patients receiving ≥1000 mL had a substantially higher incidence compared with those receiving <1000 mL. Similarly, intraoperative allogeneic blood transfusion (χ^2^ = 26.137, *p* < 0.001) and intraoperative blood loss (χ^2^ = 26.500, *p* < 0.001) were both statistically associated with hypothermia. In contrast, preoperative bowel preparation, operating room cleanliness level, and intraoperative use of cold irrigation fluid were not significantly associated with hypothermia (*p* > 0.05). Detailed results are presented in Table 2.

### 3.5. Multivariable Analysis of Risk Factors for Intraoperative Hypothermia

To identify independent risk factors, the eight variables showing statistical significance in the univariate analyses (*p* < 0.05) were entered into a multivariable logistic regression model. The dependent variable was the occurrence of intraoperative hypothermia (0 = no, 1 = yes). The eight significant variables from the univariate analysis served as independent variables and were coded in the order listed in Table 2 (assigned values 1–3 accordingly). The omnibus test of model coefficients indicated good model fit (overall χ^2^ = 256.582, overall *p* < 0.001). ASA classification, surgical approach, skin disinfection site, volume of cold intravenous fluid infusion, and surgical duration remained independently associated with intraoperative hypothermia. Specifically, a higher ASA class was associated with higher odds of hypothermia (OR = 1.408, 95% CI: 1.197–1.657, *p* < 0.001), reflecting an observed association. Compared with open surgery, superficial and minimally invasive procedures were associated with lower odds of hypothermia (OR = 0.735, 95% CI: 0.577–0.936, *p* = 0.013). Procedures involving the extremities or the thorax/abdomen/back were associated with higher odds of hypothermia (OR = 2.024, 95% CI: 1.534–2.670, *p* < 0.001). Greater volumes of cold intravenous fluids were associated with progressively higher odds of hypothermia (OR = 1.365, 95% CI: 1.140–1.633, *p* = 0.001). Longer surgical duration was also independently associated with higher odds of intraoperative hypothermia (OR = 2.014, 95% CI: 1.683–2.411, *p* < 0.001). By contrast, anesthesia type, intraoperative allogeneic blood transfusion, and intraoperative blood loss—although significant in univariate analyses—did not remain independent predictors in the multivariable model (*p* > 0.05). Detailed results are presented in Table 3.

## 4. Discussion

### 4.1. Significance of the Multicenter Clinical Investigation on Intraoperative Hypothermia in Adult Patients

The value of a multicenter collaborative investigation in clinical research lies primarily in its representativeness, generalizability, and evidential strength. This study was conducted across 12 tertiary general hospitals nationwide, encompassing major regions including Central China, East China, South China, Southwest, Northwest, North, and Northeast China, with a total sample size of 4516 cases. Such a broad coverage effectively reflects the real situations of diverse populations across regions, specialties, and surgical types in China. Compared with previous studies that were predominantly single-center and retrospective in design, the multicenter prospective design of this study not only minimizes regional bias but also enhances the external validity of the findings, rendering the conclusions more generalizable and clinically instructive. Prior to study initiation, standardized training was provided for researchers at all participating centers, and a unified “Adult Intraoperative Hypothermia Risk Assessment Scale” along with a consistent core temperature monitoring protocol was employed. These methodological standardizations ensured the rigor of data collection and evaluation, thereby reducing operator-related bias and improving the reliability of the findings. The study identified ASA classification, surgical approach, skin disinfection site, volume of cold intravenous fluid infusion, and duration of surgery as factors independently associated with intraoperative hypothermia. Although several of these factors have been reported in earlier studies, the distinctive contribution of the present investigation lies in its large-scale, multicenter, prospective design and its ability to characterize how these associations manifest across different regions, surgical specialties, and clinical practice patterns within a Chinese population, rather than implying direct causation, these results provide evidence-based signals for risk stratification in routine perioperative care, highlighting differences that may be influenced by regional practice norms, perioperative resource allocation, and patient characteristics unique to Asian populations. Through the heterogeneity of multicenter samples, the research revealed both the common patterns and regional variations in factors associated with intraoperative hypothermia across populations, providing a robust data foundation for developing more precise risk prediction tools, establishing prevention strategies, and formulating a standardized perioperative temperature management system in China. Furthermore, the prevention and control of intraoperative hypothermia involves multidisciplinary collaboration among anesthesiology, surgery, and nursing teams. The multicenter cooperative study fostered interprofessional communication and collaboration, heightened awareness of intraoperative hypothermia among healthcare professionals, and promoted consensus on its management. Collectively, these efforts contribute to improving the overall quality of perioperative management and may help reduce the incidence of intraoperative hypothermia, ultimately enhancing patient outcomes.

### 4.2. Analysis of the Characteristics of Intraoperative Hypothermia in Surgical Patients

Beyond confirming previously reported risk factors, this study provides a comprehensive overview of the distribution characteristics of intraoperative hypothermia across departments, surgical sites, age groups, sex, and BMI categories in a large Chinese cohort. These findings emphasize that the risk of intraoperative hypothermia is not uniform but varies substantially according to procedural context and patient subgroups, underscoring the importance of individualized and specialty-specific temperature management strategies.

#### 4.2.1. Analysis of Preoperative Baseline Core Temperature Characteristics

In this study, the preoperative baseline core temperature was found to be significantly associated with the occurrence of intraoperative hypothermia. Among patients with a preoperative core temperature ≤ 35.9 °C, the incidence of intraoperative hypothermia reached 85.93%, which was markedly higher than that of patients with a preoperative core temperature ≥ 36.0 °C (21.91%). This is a directly observed finding in our cohort and suggests that preoperative core temperature may reflect an individual’s thermoregulatory reserve and may serve as an early indicator for hypothermia risk stratification. In the context of routine perioperative care in many Chinese hospitals, where systematic prewarming is not universally implemented, this finding highlights preoperative baseline temperature as a particularly practical and sensitive marker for early risk stratification. A lower baseline temperature, when combined with anesthesia-induced peripheral vasodilation, redistribution of heat from the core to the periphery, and environmental factors such as surgical exposure and infusion of unheated fluids, may contribute to a more rapid decline of core temperature below the critical threshold of 36.0 °C during surgery [7,8]. These mechanisms should be interpreted as interpretive discussion rather than direct causal evidence from this observational dataset. This phenomenon may be more pronounced in elderly patients, individuals with low body mass index (BMI), chronic comorbidities, or impaired peripheral circulation, who often exhibit mild hypothermia even during the preoperative preparation stage. These patients may therefore be more susceptible to intraoperative heat loss [9,10,11]. Therefore, preoperative core temperature could be considered in perioperative risk assessment frameworks. For patients with a preoperative baseline core temperature ≤ 35.9 °C, proactive preventive interventions may be beneficial, including optimizing the operating room ambient temperature, initiating prewarming using thermal blankets at least 30 min before induction, applying forced-air warming devices, warming intravenous fluids, and maintaining continuous warming during prolonged or extensive surgeries. These recommendations are proposed clinical implications based on the observed association, not proof of efficacy within this study. Such early interventions can help elevate patients’ core temperature before anesthesia induction, thereby reducing the incidence of intraoperative hypothermia and ultimately improving perioperative safety and postoperative outcomes.

#### 4.2.2. Departmental Analysis of Intraoperative Hypothermia Incidence

The results of this study revealed significant variations in the incidence of intraoperative hypothermia among different surgical departments. The highest rates were observed in hand surgery (51.35%), emergency surgery (44.44%), and cardiovascular surgery (40.07%), while otorhinolaryngology (13.92%) and obstetrics and gynecology (16.24%) exhibited relatively lower incidences. These percentages represent the observed distribution of hypothermia in our cohort. These disparities are likely related to differences in surgical characteristics, patients’ baseline conditions, and perioperative management strategies. Hand surgeries often involve extensive exposure and tourniquet use, which may restrict local blood circulation and increase heat dissipation [12]. Emergency surgery patients often lack adequate preoperative preparation, which may increase their susceptibility to hypothermia [13]. Cardiovascular surgery is typically characterized by prolonged operative duration, large exposure areas, and substantial fluid or blood product infusion, all of which may collectively contribute to intraoperative hypothermia [3]. In contrast, otorhinolaryngologic and obstetric–gynecologic procedures are usually shorter and involve more limited exposure, which may facilitate the maintenance of normothermia [14,15]. These explanations should be viewed as interpretive hypotheses rather than causal conclusions from this observational study. These findings suggest that perioperative temperature management should be tailored according to specialty-specific characteristics.

#### 4.2.3. Analysis of Surgical Site

The results of this study demonstrated significant differences in the incidence of intraoperative hypothermia among various surgical sites. The highest incidence was observed in upper limb surgeries (35.00%), followed by thoracic (28.43%) and lower limb surgeries (27.84%). Abdominal surgeries (23.59%) exhibited a moderate incidence, while back surgeries (13.04%) had the lowest rate. This pattern is an observed result in our dataset and indicates that hypothermia risk varies by surgical site. This distribution pattern suggests that the surgical site may be an important factor associated with intraoperative thermal balance. Upper limb surgeries often require extensive exposure and tourniquet application, which may exacerbate heat dissipation [16]. Thoracic and abdominal surgeries typically involve opening of body cavities, with prolonged exposure of the surgical field and infusion of large volumes of unwarmed fluids or blood products, both of which substantially increase heat loss [17]. Lower limb surgeries, though also characterized by wide exposure, are frequently accompanied by the use of blanket or circulating warming systems during the perioperative period, partially mitigating core temperature decline [18]. In contrast, head–neck and back surgeries are generally shorter in duration, with limited exposure areas and minimal intraoperative heat loss [19]. These mechanisms are proposed interpretations and should not be taken as proof of causality. These findings suggest that the surgical site may influence intraoperative heat dissipation through multiple interacting mechanisms.

#### 4.2.4. Analysis of Age Distribution Characteristics

The results of this study demonstrated that the incidence of intraoperative hypothermia increases progressively with age. Among patients aged 18–27 years, the incidence was 16.74%, rising to 34.83% in those aged 78–87 years, and remaining high at 33.33% in patients aged ≥88 years. These findings indicate that age is a significant factor associated with intraoperative hypothermia. The underlying mechanisms are likely associated with age-related declines in thermoregulatory capacity. With advancing age, the sensitivity of the hypothalamic thermoregulatory center decreases, sympathetic nervous activity is attenuated, and shivering-induced thermogenesis is restricted [20]. In addition, reduced subcutaneous fat and impaired peripheral circulation contribute to increased heat loss, while the high prevalence of chronic comorbidities further compromises the body’s ability to maintain thermal homeostasis [20,21]. Moreover, elderly patients exhibit slower metabolism and clearance of anesthetic drugs, resulting in more pronounced suppression of central thermoregulation. Under the combined influence of surgical exposure and infusion of unheated fluids, this pharmacological vulnerability predisposes older patients to hypothermia [22]. In contrast, younger patients generally have higher metabolic rates and stronger thermogenic capacity, which enhances their resilience to surgical stress and environmental temperature fluctuations. However, prolonged operative duration or inappropriate fluid management may still place them at risk of hypothermia [23]. Therefore, Elderly patients may represent a population with increased vulnerability to intraoperative hypothermia and may benefit from targeted perioperative temperature management strategies.

#### 4.2.5. Analysis of Sex-Related Characteristics

The results of this study showed that the incidence of intraoperative hypothermia was slightly higher in male patients (24.99%) compared with female patients (22.57%), suggesting that sex may play a role in thermoregulatory differences. Given the small absolute difference, this finding should be interpreted cautiously and primarily as a descriptive observation. This phenomenon may be explained by physiological and endocrine characteristics. Women generally have a higher proportion of subcutaneous fat, which helps reduce heat loss, whereas men have greater muscle mass but relatively lower fat reserves. Consequently, under anesthesia-induced vasodilation and increased heat dissipation from surgical exposure, men are more prone to reductions in core temperature [24]. Additionally, estrogen has been shown to exert protective effects on vasoconstriction and thermogenic responses, while men lack such hormonal regulation, potentially weakening their defense against hypothermia [25]. Previous studies have also reported that men experience a more pronounced suppression of central thermoregulation after anesthesia, with faster redistribution of peripheral blood flow compared with women, which further exacerbates the decline in core temperature [24]. However, given the modest magnitude of the difference, sex alone may not constitute an independent determinant of hypothermia risk but may interact with other perioperative factors.

#### 4.2.6. Analysis of BMI Distribution Characteristics

The findings of this study revealed significant differences in the incidence of intraoperative hypothermia across patients with varying body mass index (BMI) levels. The underweight group (<18.5 kg/m^2^) demonstrated the highest incidence (30.77%), followed by the normal weight group (18.5–24.9 kg/m^2^, 24.44%) and the overweight group (25.0–29.9 kg/m^2^, 21.08%), whereas the obese group (≥30.0 kg/m^2^) exhibited the lowest incidence (16.17%). These results suggest that low BMI is a factor associated with intraoperative hypothermia, while high BMI may be associated with a lower incidence. This phenomenon can be explained by differences in heat storage and dissipation characteristics. Underweight patients, with limited subcutaneous fat, lack an effective insulation barrier, making them more susceptible to rapid core temperature loss under the combined effects of anesthesia-induced central thermoregulatory suppression, surgical exposure, and infusion of cold fluids [26]. Conversely, obese patients generally have thicker subcutaneous fat layers that provide an insulating effect, along with relatively higher basal metabolic rates and enhanced thermogenic capacity, thereby attenuating the risk of hypothermia [27]. These explanations remain interpretive and are not demonstrated as causal mechanisms within this study. It is important to note, however, that this protective effect does not imply a lower overall perioperative risk for obese patients. Obesity is frequently accompanied by reduced cardiopulmonary reserve and metabolic disorders, which may still lead to adverse outcomes if temperature management is inadequate [28]. Therefore, BMI should be considered not only as an indicator of nutritional status but also as a reference factor for hypothermia risk stratification. In clinical practice, underweight patients should receive intensified perioperative protective measures, such as preoperative warming, continuous intraoperative forced-air warming, and fluid warming. For obese patients, while maintaining vigilant temperature management, equal emphasis should be placed on optimizing cardiopulmonary and metabolic support to achieve individualized perioperative thermal management.

### 4.3. Analysis of Intraoperative Core Temperature Changes Under Different Warming Interventions

This study evaluated the effects of three different warming interventions—passive insulation, forced-air warming, and resistive heating—on intraoperative core temperature. At the onset of surgery, the core temperatures of all three groups were comparable (36.35–36.40 °C). During the early surgical period (0–210 min), a decline in core temperature was observed across all groups, with the most pronounced decrease in the passive insulation group, where some patients approached the hypothermia threshold of 36.0 °C. As surgical time progressed, the forced-air warming group and the resistive heating group exhibited gradual recovery of core temperature, maintaining higher levels compared with passive insulation. Notably, the resistive heating group showed the fastest recovery rate, surpassing the forced-air warming group in the mid-to-late phases of surgery. In contrast, passive insulation alone did not lead to a significant rebound until after approximately 450 min. It should be emphasized that this study was observational in nature, and warming interventions were selected according to routine clinical practice. The choice of warming strategy may have been influenced by surgical type, patient characteristics, procedural complexity, or centers’ preferences. Therefore, the observed differences in intraoperative core temperature trajectories should be reflected as clinical practice patterns rather than demonstrating the causal efficacy. These findings indicate associations between different warming strategies and intraoperative temperature changes, highlighting how active warming interventions are commonly applied in higher-risk or longer-duration surgeries to mitigate hypothermia. Rather than establishing comparative effectiveness, the results provide descriptive evidence of temperature management trends under routine perioperative conditions. Within this context, the results suggest that passive insulation alone may be insufficient to maintain thermal stability during prolonged or high-risk procedures, whereas active warming strategies such as forced-air warming and resistive heating are more frequently associated with improved temperature maintenance during surgery. In alignment with existing literature, the findings underscore the importance of incorporating active warming measures into perioperative temperature management protocols, particularly for patients at increased risk of hypothermia [29,30,31,32]. Future randomized or controlled studies are warranted to further evaluate the comparative effectiveness of different warming modalities.

### 4.4. Analysis of Factors Associated with Intraoperative Hypothermia in Surgical Patients

This study, through univariate and multivariate logistic regression analyses, identified several risk factors closely associated with the occurrence of intraoperative hypothermia. The results demonstrated that ASA classification, surgical approach, skin disinfection site, volume of cold intravenous fluid infusion, and duration of surgery were independently associated factors (*p* < 0.05). While these factors are generally consistent with those reported in previous studies, the present multicenter analysis further clarifies their relative importance within a heterogeneous Chinese surgical population. In particular, the skin disinfection site remained independently associated with intraoperative hypothermia after adjustment for multiple confounders, a factor that has been less emphasized in prior large-scale studies, suggesting that the extent of preoperative exposure may represent an underrecognized contributor to heat loss in routine clinical practice. Although anesthesia type, intraoperative transfusion of banked blood, and intraoperative blood loss were significantly associated with hypothermia in univariate analysis, they did not emerge as independent risk factors in the multivariate regression model. This finding suggests that, in real settings, their effects may be partly explained by more proximal variables such as surgical complexity, operative duration, and fluid management, rather than acting as isolated determinants. ① ASA classification: A higher ASA classification indicates a greater burden of comorbidities and poorer overall physiological reserve [33]. In this study, higher ASA classification was significantly associated with an increased risk of hypothermia. The underlying reason may be that patients with higher ASA grades often suffer from cardiopulmonary insufficiency, decreased metabolic capacity, and impaired thermoregulatory function [34]. In addition, under the combined effects of surgical exposure, fluid infusion, and fluctuations in ambient temperature, these patients are more susceptible to thermal imbalance [35]. Therefore, ASA classification not only reflects the overall burden of systemic disease but also indirectly indicates the vulnerability of thermoregulation. ② Surgical approach: Compared with superficial or minimally invasive procedures, open surgery involves a larger exposure field and longer operative duration, resulting in substantially increased heat loss. The use of large volumes of cold irrigation and sustained cavity opening further exacerbates the decline in core temperature. Therefore, the surgical approach is likely to be associated with the degree of heat dissipation and is an important factor associated with intraoperative hypothermia [26]. ③ Skin disinfection site: The extent of the disinfection area determines the degree of preoperative skin exposure. Particularly in regions such as the limbs, thorax, abdomen, and back, where large areas are exposed, the barrier function of the skin is compromised, leading to significant heat loss and a higher risk of hypothermia [36]. This finding suggests that, before extensive surgeries, the scope of skin preparation and exposure should be optimized to minimize unnecessary thermal loss. ④ Volume of cold intravenous fluid infusion: The temperature of infused fluids has a direct impact on patients’ core body temperature. Administration of large volumes of cold fluids accelerates heat transfer from the core to the periphery, and the greater the infusion volume, the heavier the cold load. This exerts a stronger disruption of thermal homeostasis and leads to a marked decline in core temperature [37]. The findings of this study further support the necessity of fluid warming as an integral component of perioperative temperature management. This is particularly critical in major surgeries or when large volumes of fluid infusion are anticipated, where the timely use of fluid-warming devices is essential. ⑤ Duration of surgery: The risk of hypothermia increases significantly with prolonged surgical duration. This can be attributed to the cumulative effect of intraoperative heat loss—longer surgeries not only expose patients to a low-temperature environment for extended periods but also compound the effects of continuous exposure, infusion of cold fluids, and redistribution of body heat, resulting in a progressive decline in core temperature [38,39,40]. Patients undergoing long-duration surgeries represent a key population for hypothermia prevention and control. Although anesthesia type, intraoperative transfusion of banked blood, and intraoperative blood loss were significantly associated with hypothermia in univariate analysis, they did not emerge as independent risk factors in the multivariate regression model. The possible explanations are as follows: ① Anesthesia type—different anesthetic techniques indeed influence thermoregulation. For instance, general anesthesia is often accompanied by vasodilation and suppression of thermogenic responses [41]. However, in this multicenter observational study, anesthesia techniques were selected according to routine clinical practice and centers’ protocols, and their effects may have been partially mediated by more direct or influential factors such as ASA classification, surgical approach, and duration of surgery [42,43]. Therefore, anesthesia type did not demonstrate independent statistical significance. ② Intraoperative transfusion of unwarmed banked blood can indeed lead to a decline in body temperature. However, in this study population, massive transfusion typically occurred in conjunction with major or prolonged surgeries and severe intraoperative bleeding, making it highly correlated with the volume of cold intravenous fluid infusion and duration of surgery [44]. As a result, collinearity effects in the multivariate model may have attenuated its independent contribution [45]. ③ Intraoperative blood loss undoubtedly increases heat loss from the body and often necessitates greater fluid and blood product replacement. Nevertheless, it is strongly coupled with surgical approach, operative duration, and fluid management [41,46]. In the regression model, the effect of intraoperative blood loss may have been partially accounted for by these variables, and therefore, it did not appear as an independent risk factor. In addition, several perioperative environmental and management-related factors, such as operating room ambient temperature, humidity, and the use of warmed intravenous fluids, are known to influence intraoperative thermal balance. In the present study, these variables were managed according to local clinical routines at each participating center. Although most centers followed standard recommendations for maintaining operating room temperature and selectively applied fluid-warming measures in high-risk or long-duration procedures, residual inter-center variability may still have influenced intraoperative core temperature changes. The proposed mechanisms discussed above represent plausible explanations rather than definitive causal pathways, given the observational nature of the study. Although anesthesia type, intraoperative transfusion, and blood loss did not remain independently associated in multivariate analysis, their potential contributions should still be considered in clinical temperature management. And based on the observed independent associations in this multicenter cohort, closer intraoperative core temperature monitoring and earlier escalation of active warming may be particularly warranted for patients with higher ASA grades, those undergoing open procedures or broader skin disinfection/exposure, cases requiring larger volumes of cold intravenous fluids, and surgeries of longer duration. Overall, the novelty of this study lies in providing a context-specific understanding of how established risk factors interact across regions, specialties, and perioperative practice patterns in China, thereby offering practical insights for refining individualized temperature management strategies.

Limitations of this study: Despite the adoption of a multicenter prospective design encompassing 12 tertiary general hospitals nationwide and a large sample size, this study still has certain limitations. First, although the study population was broadly sourced, all participating hospitals were large tertiary comprehensive institutions. Variations in perioperative temperature management practices among these hospitals may exist, potentially limiting the generalizability of the findings to hospitals of different levels or with different resource capacities. Second, although standardized training and unified data collection protocols were implemented, individual differences and measurement variability in intraoperative temperature management were inevitable. Environmental and management-related variables, such as operating room ambient temperature, humidity control, anesthesia maintenance techniques, and the routine use of warmed intravenous fluids, these factors were managed according to local centers’ protocols and routine clinical practice. While this approach reflects real conditions, it may have introduced residual confounding and limited the ability to fully evaluate their independent effects on intraoperative hypothermia. In addition, intraoperative hypothermia was defined in this study as a core temperature < 36.0 °C; however, the clinical manifestations of hypothermia can vary among individuals. A single temperature threshold may not fully capture its clinical significance. Future studies could integrate additional physiological parameters for a more comprehensive evaluation. Lastly, as an observational study, potential confounding factors cannot be completely excluded—for example, uncontrolled intraoperative variables or postoperative temperature changes—which may affect the extrapolation of the findings. Therefore, future research should employ more refined designs to further explore the long-term impact of these factors on intraoperative hypothermia, particularly the influence of postoperative temperature fluctuations on recovery and long-term outcomes.

## 5. Conclusions

This large-scale multicenter prospective study, conducted across 12 tertiary general hospitals in China, systematically evaluated the incidence and factors associated with intraoperative hypothermia in adult patients. The results revealed an overall incidence of 23.82%, indicating that intraoperative hypothermia is commonly observed and is associated with multiple perioperative characteristics within routine clinical practice across diverse regions of China. Specifically, preoperative baseline core temperature, duration of surgery, surgical site exposure, and volume of cold fluid infusion were identified as important factors associated with intraoperative hypothermia. Multivariate logistic regression analysis further confirmed that ASA classification, surgical approach, skin disinfection site, volume of cold intravenous fluid infusion, and duration of surgery were independently associated with intraoperative hypothermia. Notably, the incidence of hypothermia was higher in hand surgery, emergency surgery, and cardiovascular surgery departments, indicating an observed association with specific surgical specialties and suggesting that these specialties may warrant particular attention in risk assessment and temperature management. Although anesthesia type, intraoperative transfusion of banked blood, and intraoperative blood loss were significant in univariate analysis, their associations may have been indirectly mediated through other perioperative variables; nevertheless, they should not be overlooked in clinical practice. Overall, the unique contribution of this study lies in contextualizing these factors within a real Chinese cohort, thereby highlighting regional, specialty-related, and practice-based variations in hypothermia risk. These findings provide robust observational evidence to support the early identification of patients at increased likelihood of intraoperative hypothermia, underscoring the crucial role of individualized temperature management strategies in mitigating its occurrence and improving patient outcomes; however, given the observational design, all findings should be interpreted as associations rather than evidence of direct causation. Clinicians should tailor perioperative thermal management plans according to preoperative assessments and surgical characteristics and adapt existing international recommendations to local clinical contexts to optimize perioperative safety and enhance postoperative recovery, with particular consideration for closer temperature monitoring and earlier escalation of active warming in patients with higher ASA grades, longer anticipated surgical duration, broader skin disinfection or exposure, and expected larger volumes of cold intravenous fluid infusion.

## Figures and Tables

**Table 1:** Incidence of intraoperative hypothermia across different clinical characteristics.

Variable	Category	Total Surgeries (*n*)	Hypothermia Cases (*n*)	Incidence (%)
Baseline Core Temperature (°C)	≤35.9	135	116	85.93
	≥36.0	4381	960	21.91
Surgical Specialty	Otorhinolaryngology	194	27	13.92
	Orthopedics	699	150	21.46
	Obstetrics/Gynecology	271	44	16.24
	Hepatobiliary Surgery	622	144	23.15
	Thyroid/Breast Surgery	239	54	22.59
	Urology	488	110	22.54
	Thoracic Surgery	424	102	24.06
	Gastrointestinal Surgery	529	116	21.93
	Hand Surgery	37	19	51.35
	Stomatology	33	7	21.21
	General Surgery	266	80	30.08
	Neurosurgery	320	80	25.00
	Vascular Surgery	39	6	15.38
	Emergency Surgery	54	24	44.44
	Cardiovascular Surgery	282	113	40.07
Surgical Site	Head and Neck	751	165	21.97
	Thoracic	830	236	28.43
	Abdominal	2124	501	23.59
	Back	322	42	13.04
	Upper Extremity	60	21	35.00
	Lower Extremity	291	81	27.84
	Pelvis and Perineum	134	27	20.15
Age (years)	18–27	233	39	16.74
	28–37	412	68	16.50
	38–47	539	107	19.85
	48–57	1014	245	24.16
	58–67	1225	301	24.57
	68–77	900	246	27.33
	78–87	178	62	34.83
	≥88	15	5	33.33
Sex	Male	2429	605	24.99
	Female	2087	471	22.57
BMI (kg/m^2^)	<18.5	221	68	30.77
	18.5–24.9	2639	645	24.44
	25.0–29.9	1390	293	21.08
	≥30.0	266	43	16.17

All variables are defined and categorized as described in Section 2.3.

**Table 2:** Univariate analysis of risk factors for intraoperative hypothermia.

Variable	Category	Total Surgeries (*n*)	Hypothermia Cases (*n*)	χ^2^	*p*
Preoperative Bowel Preparation	None	3344	787	0.609	0.737
	Oral laxatives	898	221		
	Cleansing enema	274	68		
Anesthesia Type	Regional anesthesia	58	11	6.584	0.037
	General anesthesia	4165	978		
	Combined anesthesia (general + regional)	293	87		
ASA Classification	I–II	3238	678	55.850	<0.001
	III	1194	365		
	≥IV	84	33		
Surgical Approach	Superficial or soft tissue	1274	289	11.143	0.004
	Minimally invasive (laparoscopic, endoscopic, microscopic)	2485	571		
	Open surgery (thoracic, abdominal, pelvic)	757	216		
Operating Room Cleanliness	Ten-thousand class	2331	550	0.648	0.723
	Thousand class	918	214		
	Hundred class	1267	312		
Skin Disinfection Site	Head and neck	749	140	27.297	<0.001
	Extremities	530	166		
	Thorax/Abdomen/Back	3235	769		
Volume of Intravenous Cold Fluid (mL)	<1000	1425	231	77.862	<0.001
	1000~2000	2359	611		
	>2000	732	234		
Volume of Cold Irrigation Fluid (mL)	<1000	2844	670	0.430	0.806
	1000~2000	1173	282		
	>2000	499	124		
Intraoperative Allogeneic Blood Transfusion (U)	<2	4279	987	26.137	<0.001
	2~4	210	78		
	>4	27	11		
Intraoperative Blood Loss (mL)	<400	4280	987	26.500	<0.001
	400~800	195	73		
	>800	41	16		
Surgical Duration (h)	<2	1712	241	177.803	<0.001
	2~4	1951	521		
	>4	853	314		

All variables are defined and categorized as described in Section 2.3.

**Table 3:** Multivariate Analysis of Risk Factors for Intraoperative Hypothermia.

Variable	*β*	*SE*	Waldχ^2^	*p*	*OR*	95% CI
Type of anesthesia (1 = Regional; 2 = General; 3 = Combined)	0.556	0.376	2.184	0.140	1.743	0.834–3.644
ASA classification (1 = I–II, 2 = III, 3 = IV)	0.342	0.083	17.063	<0.001	1.408	1.197–1.657
Surgical approach (1 = Superficial/Soft Tissue Surgery; 2 = Minimally Invasive Surgery; 3 = Open Surgery)	−0.308	0.124	6.212	0.013	0.735	0.577–0.936
Skin disinfection site (1 = Head/Neck, 2 = Extremities, 3 = Thorax/Abdomen/Back)	0.705	0.141	24.870	<0.001	2.024	1.534–2.670
Volume of cold intravenous fluids infused (mL) (1 ≤ 1000, 2 = 1000–2000, 3 ≥ 2000)	0.311	0.092	11.509	0.001	1.365	1.140–1.633
Volume of transfused blood (U) (1 ≤ 2 2 = 2–4 3 ≥ 4)	0.110	0.165	0.441	0.507	1.116	0.807–1.542
Intraoperative blood loss (mL) (1 ≤ 400, 2 = 400–800, 3 ≥ 800)	0.225	0.173	1.689	0.194	1.252	0.892–1.756
Duration of surgery (h) (1 ≤ 2, 2 = 2–4, 3 ≥ 4)	0.700	0.092	58.262	<0.001	2.014	1.683–2.411

All variables are defined and categorized as described in Section 2.3.

## Data Availability

The data presented in this study are available on request from the corresponding author due to privacy concerns.

## Appendix A

Intraoperative Hypothermia Investigators (Collaborators): The Intraoperative Hypothermia Investigators (12-center Consortium) comprise researchers from 12 tertiary general hospitals across China who participated in patient enrollment, data collection, and quality control. The participating institutions and their principal investigators were as follows: Union Hospital, Tongji Medical College, Huazhong University of Science and Technology (Wuhan, Hubei; Principal Investigator: Hanqing Zhang); Henan Provincial People’s Hospital (Zhengzhou, Henan; Principal Investigator: Yanyan Li); Renji Hospital, Shanghai Jiao Tong University School of Medicine (Shanghai; Principal Investigator: Xiaomei Chen); Guizhou Provincial People’s Hospital (Guiyang, Guizhou; Principal Investigator: Donglin Mei); Guangdong Provincial People’s Hospital (Guangzhou, Guangdong; Principal Investigator: Nan Zhou); The First Affiliated Hospital of Xi’an Jiaotong University (Xi’an, Shaanxi; Principal Investigator: Yina Dong); General Hospital of Northern Theater Command (Shenyang, Liaoning; Principal Investigator: Liyuan Meng); Qilu Hospital of Shandong University (Jinan, Shandong; Principal Investigator: Li Li); Bethune Hospital of Shanxi (Taiyuan, Shanxi; Principal Investigator: Yue Guo); Qinghai Provincial People’s Hospital (Xining, Qinghai; Principal Investigator: Zhibin Wei); Sichuan Provincial People’s Hospital (Chengdu, Sichuan; Principal Investigator: Wanlin Liu); and Wuhan Central Hospital (Wuhan, Hubei; Principal Investigator: Min Liu). All collaborators contributed to patient recruitment, data verification, and site-level coordination in accordance with MDPI’s group authorship policy.
